# Establishing SSR-Based Variety Identification and Callus Regeneration Systems for the Novel *Hordeum brevisubulatum* Cultivar ‘Mengnong No. 2’

**DOI:** 10.3390/plants15081257

**Published:** 2026-04-19

**Authors:** Hui Yang, Ruijuan Yang, Yefei Liu, Xiao Han, Yaling Liu, Yuchen Li, Xintian Huang, Yuquan Gan, Cuiping Gao, Chunxiang Fu, Yan Zhao

**Affiliations:** 1 Key Laboratory of Forage Cultivation, Processing and High Efficiency Utilization, Ministry of Agriculture and Rural Affairs/Key Laboratory of Grassland Resources, Ministry of Education/Key Laboratory of Native Grass Breeding, National Forestry and Grassland Administration, College of Grassland Science, Inner Mongolia Agricultural University, Hohhot 010018, China; 2 Synthetic Biology Technology Innovation Center of Shandong Province, Qingdao Institute of Bioenergy and Bioprocess Technology, Chinese Academy of Sciences, Qingdao 262200, China; 3 Ordos Institute of Forestry and Grassland Science, Ordos City 017010, China; 4 National Technology Innovation Center for Pratacultural, Hohhot 010010, China

**Keywords:** *Hordeum brevisubulatum*, SSR-based variety identification system, callus regeneration, genetic manipulation

## Abstract

*Hordeum brevisubulatum* ‘Mengnong No. 2’ is a new forage variety developed using traditional group selection breeding techniques. It features notable advantages in plant height, tillering capacity, and overall biomass yield. However, key molecular breeding techniques such as molecular marker identification and genetic manipulation have yet to be established for this variety, limiting improvements in important traits. Consequently, we assessed the biomass of ‘Mengnong No. 2’ against ‘Mengnong No. 1’, the most widely cultivated variety in the central and western regions of Inner Mongolia, China. We report that fresh forage, dry forage, and seed yields of ‘Mengnong No. 2’ increased by 20.6%, 31.78%, and 34.35%, respectively, compared with the control variety, indicating broad prospects for its application and promotion. To enable rapid identification of ‘Mengnong No. 2’ during its promotion and to prevent production losses caused by variety admixture, we used three screened SSR primer pairs (GST25, GST37, GST127) to construct a DNA fingerprint for five *H. brevisubulatum* varieties, including ‘Mengnong No. 2’. With the percentage of polymorphic bands exceeding 95%, these profiles enabled precise identification of the ‘Mengnong No. 2’ variety. Furthermore, callus regeneration in *H. brevisubulatum* represents a bottleneck for directed molecular breeding techniques such as genetic transformation and gene editing. Accordingly, we selected the inflorescences of ‘Mengnong No. 2’ as explants and investigated the callus induction and regeneration capacity of inflorescences at different developmental stages. We found that explants at the spikelet primordia differentiation stage exhibited the highest callus induction and regeneration efficiencies, reaching 62.7% and 72.8%, respectively. The resulting embryogenic callus lines can serve as recipients for *Agrobacterium*-mediated transformation or gene gun bombardment, facilitating the development of subsequent high-efficiency genetic transformation and gene-editing systems. The SSR-based variety identification system and the highly efficient regeneration technology using inflorescence-derived callus established in this study lay a solid foundation for the development of a molecular breeding system for ‘Mengnong No. 2’.

## 1. Introduction

*Hordeum brevisubulatum* is a perennial herbaceous grass (family Poaceae) that is also commonly known as wild rye, rye grass, barley grass, and/or short-awn barley. This species is widely distributed across northeastern and northern China, Inner Mongolia, and Xinjiang, where it is important for both ecological restoration and forage [1,2]. Because its stems and leaves are tender, nutrient-rich, and palatable, it is highly favored by livestock as a high-quality forage crop [3]. It also regreens early, matures rapidly, and is highly adaptable, cold-tolerant, drought-resistant, and salt–alkali-tolerant (capable of growing in soils with a pH ranging 8.3–9.5). Because it can survive winter temperatures as low as −40 °C and has low soil-nutrient requirements, it is an excellent species to improve moderately to severely saline–alkali soils [4,5,6].

*Hordeum brevisubulatum* ‘Mengnong No. 2’ is a novel forage variety developed using traditional group-selection breeding techniques. It possesses outstanding characteristics such as tall plant stature, vigorous tillering, and overall biomass yield, and demonstrates excellent forage quality and field performance. These attributes offer broad prospects for application and promotion. Research on *H. brevisubulatum* has largely focused on stress tolerance [7], grain shedding traits [8,9,10,11], genetic mechanisms [12], gene function [1], the development of molecular markers [13], and endophytic fungi [14,15,16]. A substantial theoretical and technical foundation has been developed. However, key molecular breeding technologies for *H. brevisubulatum* ‘Mengnong No. 2’ (e.g., SSR-based variety identification system and genetic manipulation) have yet to be established, and conventional breeding methods struggle to further improve its important traits. Unlike the homozygous, self-pollinating cultivated barley, *H. brevisubulatum* is an outcrossing species with a highly heterozygous genetic background, rendering traditional population-based selection inefficient for trait fixation [17]. Moreover, seed shattering and salt-alkali tolerance are polygenic traits strongly influenced by genotype-by-environment interactions, making it difficult for conventional breeding to break unfavorable linkages and pyramid beneficial alleles. Hence, molecular marker-assisted selection and genetic transformation are indispensable for overcoming these challenges and accelerating genetic improvement.

SSR markers possess the key characteristics of high polymorphism, codominant inheritance, and locus specificity. They serve as essential molecular tools for advancing precision and efficiency in breeding and play multiple critical roles in plant molecular breeding, including the analysis of genetic diversity, identification of germplasm resources, and marker-assisted selection for target traits [18]. To enable rapid identification of *H. brevisubulatum* ‘Mengnong No. 2’ during its application and promotion and to prevent production losses caused by variety admixture, we first assessed its biomass and compared it with *H. brevisubulatum* ‘Mengnong No. 1’ (currently the most widely cultivated variety in central and western Inner Mongolia) and *H. brevisubulatum* ‘Saertu’ (which is promoted in the north-eastern region). Concurrently, fingerprint profiles were constructed for five *H. brevisubulatum* varieties, including ‘Mengnong No. 2’, to enable its precise identification.

Genetic transformation is a core technological approach in molecular breeding that enables the efficient introduction of exogenous genes conferring desirable traits, overcomes reproductive isolation between species, and addresses genotype dependency. By facilitating the targeted transfer of functional genes, the precise improvement of agronomic traits, and the shortening of breeding cycles, it provides crucial technical support for molecular breeding [19]. In the development of genetic transformation technologies, the selection of explants directly determines the induction and regeneration capacity of callus tissue and is therefore key to establishing an efficient genetic transformation system. Currently, the commonly used recipient materials for genetic transformation in grass species include seeds [20,21], young spikes [22], immature inflorescences [23], leaves [24], and stem tips [25]. Notably, Chou et al. [26] selected immature inflorescences as explants in Sorghum and achieved a callus induction rate as high as 95%. This approach is characterized by simple in vitro handling, a high rate of embryogenic callus induction, and strong regeneration capacity, with embryogenic potential significantly superior to that of immature embryos. Thomas et al. used immature inflorescences and immature embryos to induce callus formation in *Hordeum vulgare* and reported an induction rate using immature inflorescences as explants of 39.1%, which was higher than that for immature embryos [27]. Although immature inflorescences are widely recognized as excellent explants in cereals, no systematic reports on their application for plant regeneration in *H. brevisubulatum* exist. More importantly, the precise developmental stage of the inflorescence is a critical determinant of regeneration success, yet this optimal window remains undefined. Because of the minute size of *H. brevisubulatum* seeds, sampling immature embryos is particularly challenging. Therefore, we selected immature inflorescences of ‘Mengnong No. 2’ as explants and systematically analyzed their callus induction and regeneration capacities across different developmental stages. By identifying the optimal developmental window, we aim to establish a highly efficient inflorescence-derived regeneration system to lay a solid foundation for the development of genetic transformation and gene editing platforms for *H. brevisubulatum*.

## 2. Results and Analysis

### 2.1. Phenotypic and Yield Traits of H. brevisubulatum ‘Mengnong No. 2’

**Figure 1:**
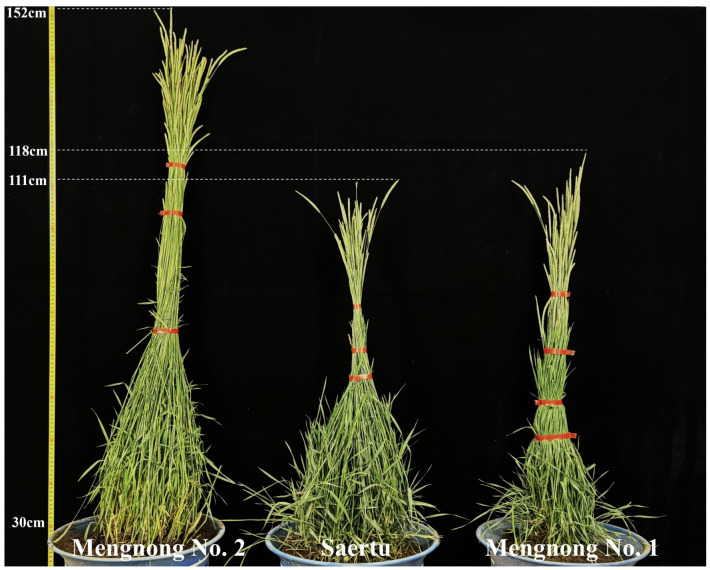
Comparison of plant height and biomass per plant among three *Hordeum brevisubulatum* varieties. Note: The 30 cm scale bar on the left indicates the height of the flower pot and is provided for visual reference. The actual plant heights (excluding pot) are as follows: ‘Mengnong No. 2’—122 cm; ‘Saertu’—81 cm; ‘Mengnong No. 1’—88 cm.

**Figure 2:**
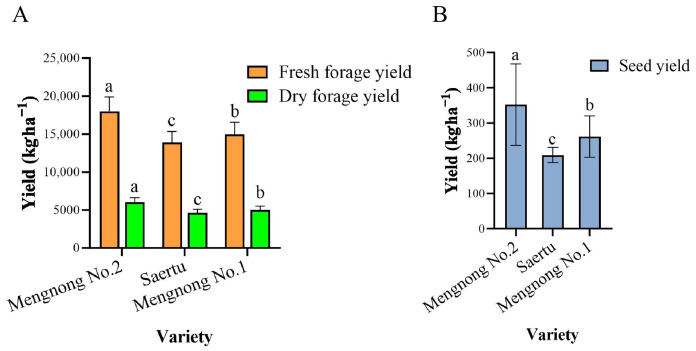
Yield performance of three *H. brevisubulatum* varieties over three years. Different lowercase letters (a, b, c) above bars denote significant differences among treatments at *p* < 0.05 (Tukey’s test), where bars sharing the same letter are not significantly different, and bars with different letters indicate significant differences. (**A**) Fresh forage yield and dry forage yield; (**B**) Seed yield.

Phenotypic analysis revealed that, compared with control varieties ‘Saertu’ and ‘Mengnong No. 1’, ‘Mengnong No. 2’ exhibited greater plant height, more vigorous tillering, and a notable advantage in total biomass (Figure 1). Three-year average yield data differed significantly (*p* < 0.05) among the three varieties in fresh forage, dry forage, and seed yields (Figure 2). Notably, fresh forage yield (18,016 kg·ha^−1^), dry forage yield (6006 kg·ha^−1^), and seed yield (352 kg·ha^−1^) of ‘Mengnong No. 2’ were significantly higher than those of the two control varieties. Compared with ‘Saertu’, these values increased by 29.58%, 42.56%, and 68.42%, respectively; compared with ‘Mengnong No. 1’, the increases were 20.6%, 31.78%, and 34.35%, respectively. These results demonstrate a substantial yield advantage (Table 1). Based on a comprehensive assessment of phenotypic and yield characteristics, ‘Mengnong No. 2’ exhibits excellent production performance. It represents a novel, high-yielding, and high-quality variety with broad prospects for application and promotion.

### 2.2. Development of an SSR-Based Variety Identification System for H. brevisubulatum

To facilitate the rapid identification of ‘Mengnong No. 2’ during its promotion, we used SSR molecular marker technology to develop DNA fingerprint profiles for this variety and four other registered *H. brevisubulatum* accessions.

**Figure 3:**
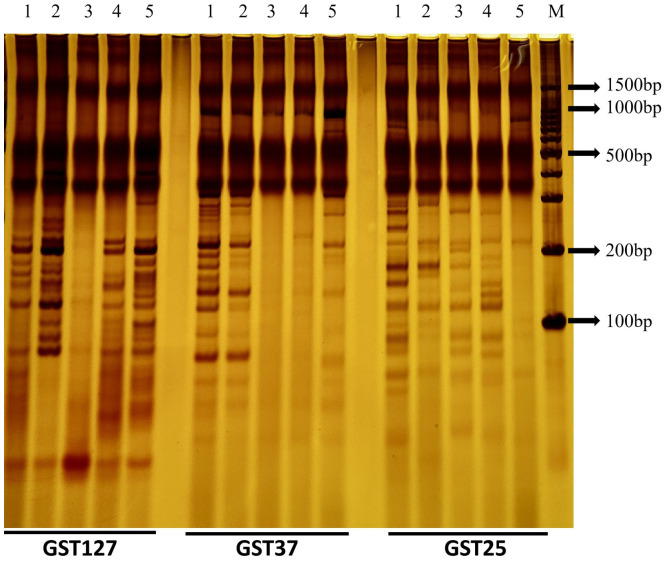
Polyacrylamide gel electrophoresis (PAGE) profile of PCR amplification products generated by primers GST127, GST37 and GST25 for *H. brevisubulatum* (varieties Ha Suhai (Lane 1), Saertu (Lane 2), Mengnong No. 2 (Lane 3), Mengnong No. 1 (Lane 4), and Hailiu (Lane 5); M represents a DNA marker).

Six pairs of previously reported SSR primers were first screened to evaluate their amplification performance across all tested materials. Among these six pairs, three (GST25, GST37, and GST127) were selected as core primers due to their clear amplification bands, good reproducibility, and locus-specific amplification. Detailed amplification characteristics of these three polymorphic primer pairs are as follows:

Primer GST25 amplified 24 distinct bands, with amplified fragment lengths concentrated between 0 and 900 bp; 23 bands (95.83%) were polymorphic. Primer GST37 amplified 22 clear bands, with amplified fragment lengths concentrated between 50 and 900 bp; 21 bands (95.45%) were polymorphic. Primer GST127 amplified 21 clear bands, with amplified fragment lengths concentrated between 50 and 400 bp; 20 polymorphic bands (95.24%) were detected (Figure 3).

**Figure 4:**
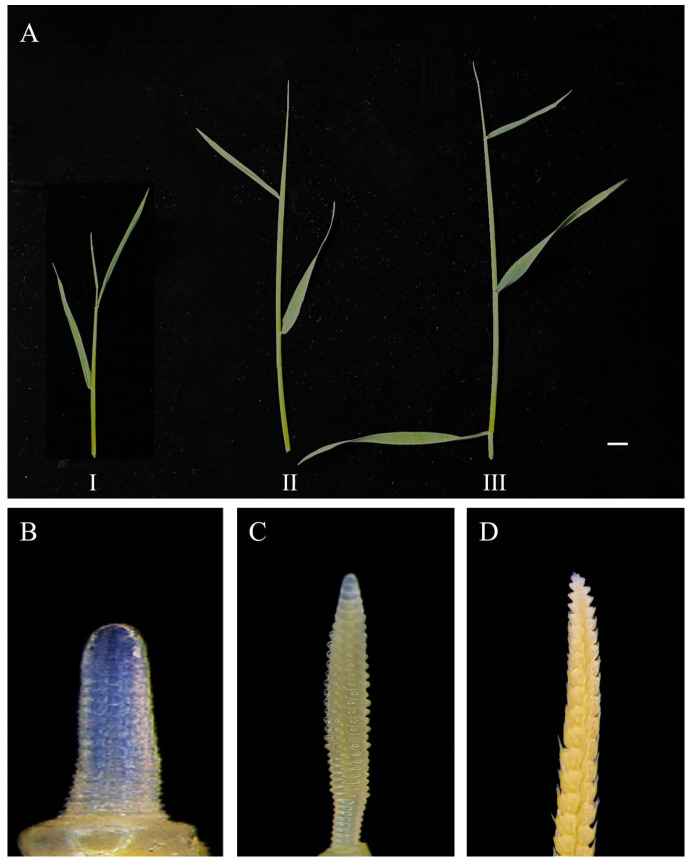
Plant morphology at different leaf developmental stages and corresponding anatomical structures of inflorescences in *H. brevisubulatum* ‘Mengnong No. 2’: (**A**) (**I**): early two-leaf one-heart stage, (**II**): two-leaf one-heart stage, (**III**): three-leaf one-heart stage, (**B**) single ridge stage (90×), (**C**) spikelet primordia differentiation stage (40×), and (**D**) stamen and pistil differentiation stage (40×). Scale bar = 1 cm.

**Figure 5:**
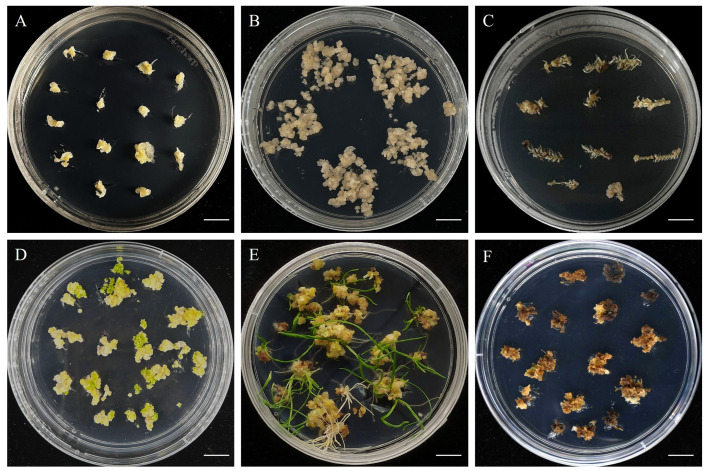
Callus induction and plant regeneration efficiency of *H. brevisubulatum* ‘Mengnong No. 2’ inflorescences at different differentiation stages: (**A**–**C**) callus induced from explants at three different inflorescence developmental stages; and (**D**–**F**) plant regeneration from the corresponding calli. Scale bar = 1 cm.

**Figure 6:**
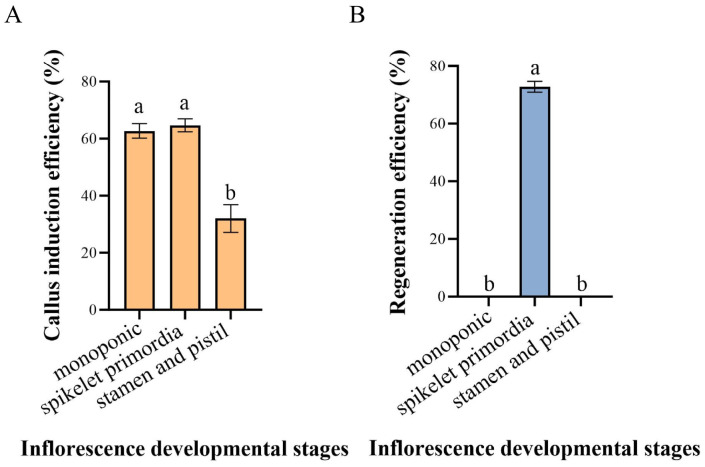
Callus induction and plant regeneration efficiency of *H. brevisubulatum* ‘Mengnong No. 2’ inflorescences at different developmental stages. Different lowercase letters (a, b) above the bars denote significant differences among treatments at *p* < 0.05 (Tukey’s test), where bars sharing the same letter are not significantly different, and bars with different letters indicate significant differences. (**A**) Callus induction rate; (**B**) Regeneration rate of callus.

Using the three selected primer pairs, DNA fingerprint profiles were successfully constructed for the five *H. brevisubulatum* varieties, including ‘Mengnong No. 2’ (Figure 4, Figure 5 and Figure 6; Table 2). The results show that each tested accession exhibited a unique banding pattern in the constructed fingerprint profiles, enabling complete discrimination among all materials. Specifically, distinct polymorphic band combinations generated by these three SSR primers allowed for the rapid and accurate identification of ‘Mengnong No. 2’.

### 2.3. Development of a Callus Regeneration System for H. brevisubulatum ‘Mengnong No. 2’

We used immature inflorescences from different developmental stages of *H. brevisubulatum* ‘Mengnong No. 2’ as explants. Sampling periods were determined based on external plant morphology, and the corresponding stages of internal differentiation within the immature inflorescences were observed (Figure 4). Observations commenced on 18 April, when plants were in the early two-leaf-one-heart stage (Figure 4(AI)). At this time, the immature inflorescences obtained by dissection were in the single ridge stage (Figure 4B), appearing as short, stout, blunt cones with a ring-like protrusion forming from the base upwards. Approximately 10 d later, plants were in the late two-leaf-one-heart stage (Figure 4(AII)). Plant height increased significantly compared with the early two-leaf-one-heart stage, and the corresponding immature inflorescences had entered the spikelet primordia differentiation stage (Figure 4C), with regularly arranged spikelet primordia protrusions appearing on the surface. At ~7 d, plants were at the three-leaf-one-heart stage (Figure 4(AIII)), corresponding to the complete differentiation of the spikelet structure of the immature inflorescences, which had entered the stage of stamen and pistil differentiation (Figure 4D), with the internal stamen and pistil primordia beginning to differentiate.

When immature inflorescences from the three aforementioned stages were inoculated onto callus induction medium (MS5), significant differences occurred in callus induction among explants at different stages. Following inoculation, explants from immature inflorescences at the single ridge stage gradually swelled and formed a pale yellow and translucent white, glass-like callus (Figure 5A). The callus structure was heterogeneous, predominantly consisting of loose, water-soaked, non-embryogenic callus, with an average induction efficiency of 62.7%. Following inoculation of immature inflorescences at the spikelet primordia differentiation stage, pale yellow, granular, densely structured embryogenic callus was formed (Figure 5B), with an average induction efficiency of 65.0%. This type of callus both grows rapidly and possesses strong regenerative capacity. When immature inflorescences at the stamen and pistil differentiation stage were inoculated, the resulting callus exhibited a loose structure, a severe water-soaked appearance, and high levels of browning (Figure 5C), with an average induction efficiency of only 32.0%. This type of callus showed no regenerative potential and was severely browned, with signs of senescence and necrosis.

After transferring the induced callus to regeneration medium (MSBK), significant differences in regeneration efficiency occurred among calli derived from immature inflorescences at different stages of differentiation. Callus derived from the single ridge stage could differentiate normally into green bud points on MSBK, but the buds did not continue to elongate upwards (Figure 5D), and the average differentiation efficiency was 0 (Figure 6). Embryogenic callus derived from the spikelet primordia differentiation stage could differentiate normally on MSBK to produce green bud points, which further developed into complete regenerated plants (Figure 5E), with an average differentiation efficiency of 72.8% (Figure 6). Regenerated plants grew vigorously with well-developed root systems and a 100% average rooting efficiency. Callus derived from the stamen and pistil differentiation stage showed almost no differentiation into bud points on MSBK, and the callus exhibited severe browning and senescence (Figure 5F); the differentiation efficiency was 0% (Figure 6). Therefore, explants derived from the spikelet primordia differentiation stage exhibited the highest callus induction efficiency and callus regeneration capacity.

## 3. Discussion

### 3.1. Yield Advantages and Promotion Potential of the Novel H. brevisubulatum Variety ‘Mengnong No. 2’

Compared with the main cultivated variety ‘Mengnong No. 1’, ‘Mengnong No. 2’ showed significant increases in fresh forage, dry forage, and seed yields (by 20.6%, 31.78%, and 34.35%, respectively). The superior yield of ‘Mengnong No. 2’ may be attributed to its taller plant stature and robust tillering capacity. In forage grasses, seed yield is primarily determined by the number of fertile reproductive stems (i.e., effective seed heads) derived from tillers [28], which likely explains the yield advantage observed in this cultivar. These values are substantially higher than the production performance of the locally dominant variety and demonstrate the superior yield traits and great application and popularization potential of this new cultivar. Central and western Inner Mongolia are located in an ecologically fragile region characterized by widespread saline–alkali land, low soil fertility, and a prominent contradiction between forage supply and demand. The high biomass of ‘Mengnong No. 2’ provides abundant high-quality forage for the development of local animal husbandry, effectively eases the forage–livestock imbalance, and has important practical significance for the restoration and improvement of saline–alkali land vegetation, enhancing regional land-use efficiency and maintaining the stability of grassland ecosystems. However, the large-scale promotion and application of elite forage cultivars often face challenges from genetic contamination and variety admixture. This requires the establishment of efficient and accurate molecular identification systems and a stable and efficient genetic transformation and regeneration system, providing solid technical support for variety protection, germplasm innovation, and further molecular breeding of *H. brevisubulatum*.

### 3.2. The Value of SSR Molecular Markers in the Identification of H. brevisubulatum Varieties

Because of their rich polymorphism, codominant inheritance, locus specificity, and excellent stability and reproducibility, SSR molecular markers have become an important technical tool for variety identification, germplasm evaluation, and molecular marker-assisted breeding [18]. Among grass forage species, SSR markers have been widely applied to the identification of varieties and to genetic diversity analysis of species such as *H. vulgare* L. var. *nudum* Hook. f. [29], *H. spontaneum* [30], and *H. vulgare* [31,32]. However, research on SSR molecular markers for *H. brevisubulatum*, an important salt-tolerant, perennial, high-quality grass in northern China, is relatively limited. Although the entire chloroplast genome of *H. brevisubulatum* has been sequenced, species-specific SSR loci have been identified, and their potential for molecular marker development has been confirmed [1], an SSR-based molecular identification system for ‘Mengnong No. 2’ has yet to be established. A lack of core markers directly applicable to the specific identification of this variety makes it difficult to meet the practical demand for rapid and accurate variety identification in large-scale production and promotion.

Traditional morphological identification is often hindered by environmental factors and developmental stages, resulting in poor stability of identification results [33,34]. In contrast, our SSR-based DNA fingerprinting system provides a stable, reliable, and efficient method for identifying ‘Mengnong No. 2’ that is unaffected by environmental interference. Using only three pairs of highly polymorphic core primers (GST25, GST37, and GST127), a polymorphism detection rate > 95% can be achieved, enabling the clear differentiation of ‘Mengnong No. 2’ from closely related varieties. This system is efficient, economical, and highly practical and is important for ensuring seed purity, preventing variety admixture, enabling rapid testing, and promoting standardized dissemination of ‘Mengnong No. 2’. While our molecular identification system can meet the basic requirements for current variety identification, the number of SSR markers used remains relatively limited. Future research could develop more highly specific SSR markers, conduct association analyses between markers and important agronomic traits, and identify functional markers closely linked to traits such as high yield, salt tolerance, and stress resistance, thereby providing more comprehensive technical support for molecular marker-assisted selection breeding of ‘Mengnong No. 2’.

### 3.3. The Effect of Explant Selection on the Establishment of a Callus Regeneration System for H. brevisubulatum

An efficient and stable regeneration system is a prerequisite for conducting genetic transformation and gene-editing research. The selection of explants is a key factor influencing the efficiency of callus induction and plant regeneration [35]. In grass species, to establish efficient regeneration systems, attempts have been made to utilize various explants as recipient materials for genetic transformation, including root apical meristems, stem segments, leaves, mature seeds, immature inflorescences and anthers [36,37,38].

Establishing a tissue culture system for *H. brevisubulatum* has traditionally been difficult due to its recalcitrant nature in ex vivo culture, as reflected by the genetic and epigenetic instabilities in tissue culture-derived regenerants [39] and the documented recalcitrance of the *Hordeum* genus [40]. In *H. vulgare*, immature embryos are the preferred explants [41,42]; however, the immature embryos of *H. brevisubulatum* are extremely small, making precise isolation technically prohibitive and presenting a technical hurdle. To resolve this, we used immature inflorescences as explants and overcame problems with obtaining immature embryos. Notably, a critical gap in the tissue culture of *H. brevisubulatum* has been the lack of precision regarding the optimal explant sampling stage. While Li et al. established a regeneration system using young inflorescences, they did not define a precise developmental stage [39]. Similarly, Liu et al. developed a tissue culture system without emphasizing sampling timing [43]. Our study directly addresses this gap by identifying the spikelet primordia differentiation stage as the optimal developmental window for callus induction and plant regeneration. Tissues at the spikelet primordia differentiation stage are characterized by a delicate cellular structure with moderate differentiation status and favorable endogenous hormone levels. These attributes enable synergistic interaction with exogenously supplied 6-BA and KT, thereby substantially enhancing callus differentiation and plant regeneration, ultimately resulting in optimal culture performance. Biologically, cells at this developmental stage are highly meristematic and possess peak totipotency, making them highly responsive to dedifferentiation stimuli. Younger explants often suffer from necrosis, whereas inflorescences harvested too late exhibit reduced morphogenic competence due to advanced cellular differentiation. We ultimately achieved a regeneration efficiency of 72.8%. For this recalcitrant species, this represents a significant technical breakthrough and lays an important foundation for establishing genetic transformation and gene-editing systems.

In conclusion, our integration of molecular identification and tissue culture advancements has established a comprehensive technical framework for the genetic improvement of ‘Mengnong No. 2’. The highly regenerative embryogenic calli, derived from optimally selected inflorescences, serve as ideal recipient materials for Agrobacterium-mediated transformation or biolistic bombardment. As a clear next step, we propose to knock out the *Btr1* and *Btr2* genes via CRISPR/Cas9, which are known to control seed abscission zone formation and seed shattering in *Hordeum* species, with the goal of reducing grain loss and improving harvest efficiency in ‘Mengnong No. 2’ to accelerate the molecular breeding process of elite forage grasses.

## 4. Materials and Methods

### 4.1. Materials

*Hordeum brevisubulatum* ‘Mengnong No. 2’ and four other registered and approved varieties (‘Hasuhai’, ‘Saertu’, ‘Mengnong No. 1’, and ‘Hailiu’) were examined (Table 3). Six SSR primer pairs [33] were synthesized by Guangzhou Ruibo Xingke Biotechnology Co., Ltd. (Guangzhou, China) (Table 4).

### 4.2. Test Site Overview

The Inner Mongolia Agricultural University pasture research station in Hohhot City, Inner Mongolia (40°48–50′ N, 111°41–45′ E) is situated in a plain area at ~1000 m altitude. The site experiences a temperate continental monsoon climate, with an annual average temperature of 3–8 °C, and annual precipitation of 300–400 mm (concentrated mainly in summer). The soil is classified as sandy chestnut calcareous soil (Calcic Kastanozem) with a pH of 7.5 and moderate fertility. During the sampling period, mean daily temperature ranged from 18 to 22 °C, the photoperiod followed natural daylight, and soil moisture was maintained at 60–70% of field capacity.

### 4.3. Methods

#### 4.3.1. Field Experiment Design and Trait Determination

Field experiments were conducted at the Pasture Research Station of Inner Mongolia Agricultural University, Hohhot, Inner Mongolia, China. Test materials, including *Hordeum brevisubulatum* ‘Mengnong No. 2’ and two other control varieties, were sown in autumn 2018, and phenotypic traits were monitored continuously for three consecutive years from 2019 to 2021. A completely randomized block design with three replications was adopted, and uniform field management was implemented across all plots; forage yield plots were 20 m^2^ with 30 cm row spacing, while seed yield plots were also 20 m^2^ with 50 cm row spacing, both sown via artificial ditching and drill seeding at a depth of 2–3 cm. Yield traits were determined at forage maturity each year: fresh forage yield was weighed immediately after mowing and converted to kg·ha^−1^, hay yield was calculated after oven-drying samples to constant weight, and seed yield was measured after threshing and air-drying to stable moisture content, with all values presented as the mean of three replications.

#### 4.3.2. Genomic DNA Extraction and Analysis

Ten plants were randomly selected from each of the five varietal populations, yielding a total of 50 samples. Genomic DNA was extracted using the 2 × CTAB method. DNA concentration and quality were assessed via 1% agarose gel electrophoresis and a micro-UV spectrophotometer. Equal volumes of genomic DNA from each of the 10 individuals per variety were pooled to form a variety-mixed DNA pool as the template for PCR amplification. Pooled DNA was diluted to 30 ng μL^−1^ based on the required amount and stored at 4 °C for future use. Remaining samples were stored at −20 °C.

#### 4.3.3. PCR Amplification

The total reaction volume for PCR amplification was 20 μL, comprising 1 μL of 30 ng/μL DNA template, 1 μL of each primer (forward and reverse), 10 μL of 2× Taq PCR Mix, and 7 μL of ddH_2_O. PCR amplification involved pre-denaturation at 94 °C for 3 min; 30 cycles of denaturation at 94 °C for 30 s, annealing at 30 s (based on primer annealing temperature), an extension at 72 °C for 1 min; and a final extension at 72 °C for 5 min. PCR products were stored at −20 °C until further use.

#### 4.3.4. Primer Screening for SSR Analysis

Six previously published SSR primer pairs were initially evaluated to identify optimal markers for accurate variety identification. The final three primer pairs (GST25, GST37, GST127) were selected according to three predefined criteria: (1) the ability to consistently amplify clear, stable, and reproducible bands across all five tested *H. brevisubulatum* accessions in at least three independent PCR replicates; (2) the presence of distinct polymorphic loci capable of discriminating among varieties; and (3) the absence of non-specific amplification products or primer-dimer artifacts, as verified by agarose gel electrophoresis. The remaining three primer pairs were excluded due to poor amplification and inability to discriminate among the five varieties.

#### 4.3.5. Electrophoresis and Staining

PCR amplification products were analyzed by 8% non-denaturing polyacrylamide gel electrophoresis. Each sample (6 μL) was loaded directly onto the gel. The DL100 Marker (Beijing Tiangen Company (Beijing, China)) was used as a control. Electrophoresis was performed at a constant 130 V for 30 min for pre-running, followed by 80 min at 130 V. After removal, the gel was placed in a fixing solution (10 mL 10% ice-cold acetic acid + 20 mL anhydrous ethanol + 170 mL distilled water) for 30 min, then a staining solution (0.4 g silver nitrate + 200 mL distilled water) for 15 min, followed by two rinses in distilled water (first wash 60 s, second wash 50 s). Finally, the gel was placed in a staining solution (3.2 g sodium hydroxide + 200 mL distilled water + 2.16 mL 37% formaldehyde) for ~2–3 min until bands became clearly visible. All steps, including fixation, staining, and washing, were performed on a shaking platform. Images were captured and then electronically scanned.

#### 4.3.6. Inflorescence Observation, Sampling, and Callus Induction Culture

Experiments were performed in 2024. Observations began at the regreening stage. Field observations were made every 2–3 d, each time randomly selecting 20 tillers that showed roughly uniform growth. The timing corresponding to each inflorescence differentiation stage was recorded at 9:00 a.m. daily, and photographs were taken to document tiller external morphology. For internal observation, tillers were dissected under a Leica DMC 5400 stereomicroscope to examine the developmental progression of inflorescence; the specific stage of spikelet differentiation was determined based on morphology.

Young inflorescences at the targeted developmental stages were collected and transferred to a laminar flow hood for surface sterilization. Explants were treated with 75% alcohol for 3 min, then air-dried to remove residual alcohol on the surface. After sterilization, young spikes were inoculated onto MS5 induction medium, which contained 4.43 g L^−1^ MS powder, 30 g L^−1^ sucrose, 5 mg L^−1^ 2,4-D and agar, with the pH adjusted to 5.95. Cultures were maintained at 22 °C in complete darkness. The callus induction rate was calculated after 20 d using Formula (1):

callus induction rate (%) = (number of young spikes producing callus/total number of inoculated young spikes) × 100%(1)

During callus culture, subculture was performed every 15 d. After 30–45 d of culture, calli were transferred to MS2 subculture medium composed of 4.43 g L^−1^ MS powder, 30 g L^−1^ sucrose, 2 mg L^−1^ 2,4-D and agar at pH 5.95. Following two to three rounds of subculture, high-quality embryogenic calli were selected and transferred to MSBK regeneration medium. This medium consisted of 4.43 g L^−1^ MS powder, 30 g L^−1^ sucrose, 0.5 mg L^−1^ 6-BA, 2 mg L^−1^ KT, and agar, pH 5.95. Regeneration cultures were maintained at 22 °C under a 14 h light/10 h dark photoperiod with a light intensity of 2000 lux. The regeneration rate was calculated after 30 d of culture using Formula (2):

regeneration rate (%) = (number of calli producing buds/total number of calli transferred to regeneration medium) × 100%(2)

#### 4.3.7. Data Processing and Analysis

Experimental data and images were organized using Microsoft Excel 2013 and PowerPoint 2024 software. Graphs and visualizations were generated with GraphPad Prism 10.1.2 software for statistical plotting and data presentation.

## 5. Conclusions

We established two key technical systems for *H. brevisubulatum* ‘Mengnong No. 2’. The first is an SSR-based molecular marker-assisted identification system. Using three pairs of SSR primers (GST25, GST37, GST127) identified through screening, a fingerprint profile comprising five *H. brevisubulatum* varieties was constructed, with a polymorphic band percentage > 95%. This enabled accurate identification of ‘Mengnong No. 2’. Second, a highly efficient regeneration system was established using immature inflorescences at the spikelet primordia differentiation stage as explants, achieving a regeneration rate of 72.8%. Embryogenic callus lines obtained from this process can serve as recipients for *Agrobacterium* infection or gene gun bombardment, providing ideal material for the development of genetic transformation and gene-editing systems. Establishing these two key technologies provides important technical support for the targeted improvement of important traits in *H. brevisubulatum* ‘Mengnong No. 2’, the identification and validation of functional genes, and the large-scale promotion of superior varieties, and lays a solid foundation for the construction of its molecular breeding system.

## Figures and Tables

**Table 1:** Three-year average yield of three *H. brevisubulatum* varieties.

Variety (Strain) Name	Fresh Forage Yield (kg·ha^−1^)	Dry Forage Yield (kg·ha^−1^)	Seed Yield (kg·ha^−1^)
Mengnong No. 2	18,016 ± 1887	6606 ± 629	352 ± 115
Saertu	13,903 ± 1435	4634 ± 478	209 ± 22
Mengnong No. 1	14,939 ± 1608	5013 ± 528	262 ± 59

Note: Values are means ± standard deviation (SD) of three-year field trials.

**Table 2:** Digital DNA fingerprint profiles of five *H. brevisubulatum* varieties based on three SSR primers.

Lane	Sample	Primer GST25	Primer GST37	Primer GST127
1	Ha Suhai	101110101011001010111111	1101011011001111111101	010001010010010101001
2	Saertu	000001000001001100100000	1010100100010100100101	010110100101101001111
3	Mengnong No. 2	000000010001101000101100	1000000000000000000000	010000100000000000000
4	Mengnong No. 1	000000000101011011101100	1000000000100000100000	010000101000000101011
5	Hailiu	010000000001000000000000	1110000100010000100010	111000101010010011111

Note: In the digital fingerprint matrix, ‘1’ indicates the presence of an amplified polymorphic band at the corresponding locus, and ‘0’ indicates the absence of an amplified polymorphic band.

**Table 3:** *H. brevisubulatum* fingerprint profile test materials.

Code	Variety (Strain)	Source	Grass Variety Approval Committee
1	Ha Suhai	Inner Mongolia Grass Seed Industry Technology Innovation Center of Mengcao Ecological Environment (Group) Co., Ltd. (Hohhot, Chian)	Inner Mongolia Autonomous Region
2	Saertu	Northeast Agricultural University	National Forestry and Grassland Administration
3	Mengnong No. 2	Inner Mongolia Agricultural University	National Forestry and Grassland Administration
4	Mengnong No. 1	Inner Mongolia Agricultural University	Inner Mongolia Autonomous Region
5	Hailiu	Inner Mongolia Agricultural University	Inner Mongolia Autonomous Region

**Table 4:** Basic primer data for SSR markers.

No.	Primer Name	Forward Primer (5′ → 3′)	Reverse Primer (5′ → 3′)	Annealing Temperature (°C)
1	GST 1	GGTGCTGTTTGTTTGTCT	GAATGAAAGTTGCGGGTT	45
2	GST 4	GTGCCCTATCAAGATTACG	TTCATCGGGACACCTTTT	50
3	GST 25	AAGTGCCAACTAGGAGTT	CATCACCATTTTACAGGG	50
4	GST 37	GGTTTCAGACACCTGTTTA	CCAATGTATGTATCTAGCAAG	50
5	GST 99	AGTAGCTAAAAATGAGCAGGCT	CTTGTTGCAAACACTAGGGTAA	50
6	GST 127	GGAGGGGATAAAACTAAAGGT	ATCGTGCCAAATCAAGAATAC	55

## Data Availability

The original contributions presented in the study are included in the article; further inquiries can be directed to the corresponding authors.
